# Seagrass sedimentary deposits as security vaults and time capsules of the human past

**DOI:** 10.1007/s13280-018-1083-2

**Published:** 2018-08-20

**Authors:** Dorte Krause-Jensen, Oscar Serrano, Eugenia T. Apostolaki, David J. Gregory, Carlos M. Duarte

**Affiliations:** 1 Red Sea Research Center (RSRC), King Abdullah University of Science and Technology, Thuwal, 23955-6900 Saudi Arabia; 2 School of Science, Centre for Marine Ecosystems Research, Edith Cowan University, Joondalup, WA Australia; 3 Institute of Oceanography, Hellenic Centre for Marine Research, PO Box 2214, 71003 Heraklion, Crete, Greece; 4 Department of Conservation and Natural Science, The National Museum of Denmark, Copenhagen, Denmark; 5 Department of Bioscience, Aarhus University, Vejlsøvej 25, 8600 Silkeborg, Denmark

**Keywords:** Conservation, Cultural heritage, Ecological service, Seagrass, Sediment deposits

## Abstract

Seagrass meadows form valuable ecosystems, but are considered to have low cultural value due to limited research efforts in this field. We provide evidence that seagrass deposits play a hitherto unrealized central role in preserving valuable submerged archaeological and historical heritage across the world, while also providing an historical archive of human cultural development over time. We highlight three case studies showing the significance of seagrass in protecting underwater cultural heritage in Denmark, the Mediterranean and Australia. Moreover, we present an overview of additional evidence compiled from the literature. We emphasize that this important role of seagrasses is linked to their capacity to form thick sedimentary deposits, accumulating over time, thereby covering and sealing submerged archaeological heritage. Seagrass conservation and restoration are key to protecting this buried heritage while also supporting the role of seagrass deposits as carbon sinks as well as the many other important ecosystem functions of seagrasses.

**Electronic supplementary material:**

The online version of this article (10.1007/s13280-018-1083-2) contains supplementary material, which is available to authorized users.

## Introduction

Recognition of the ecological functions and societal services provided by seagrass meadows has grown rapidly, propelled by the realization of their role as intense “Blue Carbon” sinks with applications to climate change mitigation and adaptation (Duarte et al. 2013) and their role in supporting biodiversity and fisheries (Ruiz-Frau et al. 2017). Cultural services (related to research/education, recreation/tourism, cultural heritage/identity) are also included among the recognized services of seagrasses (Ruiz-Frau et al. 2017), but recent global assessments still rank these services low relative to those of other ecosystems, with seagrasses e.g. supplying only 0.3% of the cultural value provided by coral reefs (Costanza et al. 2014). Economic valuation of seagrass services often ignores cultural ones (Dewsbury et al. 2016), but the perception that seagrass ecosystems have low or negligible cultural value may also derive from a paucity of analyses rather than a thorough assessment, as shown by a recent review that identified seagrass as the marine habitat whose cultural services have received the least research attention (Martin et al. 2016).

Here, we contend that previous assessments of cultural services of seagrass ecosystems, including those listed above, may have greatly overlooked their contribution. We provide evidence that seagrass meadows play a hitherto unrealized pivotal role in the preservation of valuable underwater cultural heritage across the world by covering and sealing coastal archaeological deposits, thereby serving as security vaults. We also highlight that seagrass sedimentary deposits may contain an archive of human cultural development through time by accumulating traces of human culture, thereby serving as time capsules of the human past. We support our argument by three main case studies showing the significance of seagrass in preserving submerged archaeological and historical heritage in Denmark, the Mediterranean and Australia. Moreover, we provide an overview of additional evidence from other geographical areas compiled from the literature. We emphasize that this hitherto neglected cultural service is closely linked to the capacity of seagrass meadows to produce thick sedimentary deposits. Seagrass deposits hence link a variety of ecosystem services as they also underpin the role of seagrass meadows as valuable Blue Carbon ecosystems mitigating climate change through the sequestration of carbon dioxide (Duarte et al. 2013). We note, however, that the wide array of morphology and life history traits displayed among seagrass species entails differences in their capacity to accumulate sediments (Carruthers et al. 2007), and thereby to bury and preserve archaeological remains under anoxic conditions. In addition, some seagrass meadows grow over very shallow sediments and do not seem to be able to accumulate the thick deposits required to bury and preserve archaeological remains.

## Seagrass sediment deposits as security vaults of underwater archaeological heritage

More than 100 million years ago, vascular plants inhabiting the intertidal zone adapted to live in the sea, giving rise to seagrasses. The first seagrass fossils (*Posidonia*) date back to the Cretaceous, around 120 million years ago (Blondel 2010). Seagrasses are key species found in shallow waters around the world (Orth et al. 2006) down to 90 m depth at maximum (Duarte 1991), and the majority of seagrass ecosystems grow in sheltered coastal environments where coastal communities have primarily settled over time. Already by the end of the 20th century, about 40% of the human population inhabited the coastal zone (Independent World Commission on the Oceans 1998) and the trend is increasing (Neumann et al. 2015), providing evidence of the potential interactions between human activities and seagrass meadows through time. Humans spread through the world from Africa about 60 000 years ago, using the coastal zone as a corridor to reach Australia, and later on followed the coastline once again to colonize America (Stringer 2000; Oppenheimer 2009). The artefacts left behind by these coastal communities were flooded following the gradual 120 m sea-level rise occurring over the last 20 000 years (Lambeck and Chappell 2001) and subsequently covered by sediments allowing the growth of seagrass meadows that overgrew and protected this heritage. While many of the coastal areas that hosted early human settlements are now located at water depths too deep for modern seagrass meadows to thrive, it is likely that past meadows growing in those areas as well as the deepest-growing extant meadows, may have played a role in the initial burial of these sites. The archaeological artefacts embedded within sedimentary layers below seagrass meadows range from ships (wrecks) to prehistoric fishing and other flint tools, textiles, weapons and ceramics ((Fischer 2011; Abelli et al. 2016); Table S1). Such items have been discovered when excavating ancient coastal plains subsequently flooded and covered by seagrass (Fischer 2011; Soter and Katsonopoulou 2011) or when the artefacts became exposed following seagrass loss and sediment erosion (Fischer 2011; Gregory and Manders 2016).

Due to their combined high productivity, capacity to attenuate waves and currents and to trap and bind particles, seagrass meadows raise the seafloor (Duarte et al. 2013). A recent survey reported an average difference in short-term sediment elevation rates between seagrass-vegetated and unvegetated areas of 31 mm per year with large variability between meadows (Potouroglou et al. 2017). The persistence of seagrass rhizomes, roots and leaf sheaths through time, due to the anoxic conditions prevailing in these deposits and the recalcitrant nature of seagrass remains, leads to the formation of sediment deposits of varying thickness with long-term sediment accumulation rates (SAR) ranging from 0.6 to 5 mm year^−1^ (Marbà et al. 2015; Serrano et al. 2016a), keeping in mind that the SAR in surface sediments may be overestimated due to biomixing especially in non-*Posidonia* meadows (Johannessen and Macdonald 2016). While seagrass meadows in general have the potential to stabilize sediments, protect underlying archaeological layers and serve as historical archives, the thick seagrass deposits may in addition embed archaeological artefacts.

The capacity of seagrass meadows to bury and preserve archaeological artefacts is influenced by interactions of biological factors such as growth pattern, meadow productivity, cover and density, chemical factors such as recalcitrance of seagrass debris and physical factors such as water depth, hydrodynamic energy and soil accumulation rates (Serrano et al. 2016b). Large and long-living seagrass meadows of the genera *Posidonia* and *Thalassia* can build organic-rich deposits several meters in thickness in certain habitats (Mateo et al. 1997; Lo Iocano et al. 2008; Duarte et al. 2013), while opportunistic and/or low biomass seagrass meadows of the genera *Halophila* and *Zostera* do not build similarly thick sediments. Seagrass meadows inhabiting areas with e.g. low hydrodynamic activity, fairly rapid sediment deposition and high sedimentary organic carbon content with low oxygen concentrations should be seen as a suitable habitat for preservation of archaeological heritage. In highly depositional environments, even meadows formed by small and fast-growing species, can exhibit enhanced capacity for sediment accumulation (Potouroglou et al. 2017). Hence, linking aspects of seagrass habitat, physical aspects of the environment and seagrass life history provides a context for understanding their potential role in preserving archaeological remains.

Permanency of seagrass deposits is obviously a key requirement for the protection of archaeological remains by seagrasses, and the many case studies reported below document situations where this requirement has been fulfilled. However, various climate- and human-induced environmental processes have been impacting seagrass during the Late Holocene, and the study of Posidonia mats in the NW Mediterranean Sea revealed effects of factors such as enhanced continental soil erosion and eutrophication of coastal waters since Roman-Medieval times (López-Merino et al. 2017) even though losses of seagrasses have only been reported since the 20th century. Major losses have occurred due to events such as the wasting disease, which extirpated most of the north-Atlantic eelgrass populations in the 1930s (e.g. Rasmussen 1973) and worldwide mainly due to human impacts accelerating in the late 20th century (Orth et al. 2006; Waycott et al. 2009). Such losses have led to exposure of archaeological remains (Fischer 2011) and major changes in the seafloor, especially in exposed settings even though roots and rhizomes may still exert a stabilizing effect years after seagrass decline (Rasmussen 1973). A recent study also demonstrated that seagrass loss triggers the erosion of historic carbon deposits while revegetation effectively restores seagrass carbon sequestration capacity (Marbà et al. 2015).

The age of sedimentary deposits under extant seagrass meadows can be up to 6000 years (Lo Iocano et al. 2008). These deposits are now receiving significant attention because of the large organic carbon stocks contained therein, ranging between 4.2 and 8.4 Pg of organic carbon within the top meter of seagrass soils worldwide (Fourqurean et al. 2012). However, the role of seagrass deposits in preserving underwater archaeological heritage (Fischer 2011; Polzer 2012) and recording human development through time remains unaccounted for in assessments of the cultural services provided by seagrasses despite the link between seagrass ecology and marine archaeology being implicitly made already in 1969 when seagrass debris was successfully used to determine the period when a ship sunk in Malta (Frost 1969). This shipwreck was buried below a 4-m-thick *P. oceanica* mat, and was estimated to have been buried 1100 cal. year BP, as identified, probably for the first time, by radiocarbon dating of the seagrass mat (Frost 1969), yielding the earliest estimate of seagrass sediment accretion rate of about 4 mm year^1^. However, once removed by excavation, seagrasses are often not capable to re-establish leading to the exposure of the artefacts, compromising their preservation (Godfrey et al. 2005).

## Case studies of seagrasses as security vaults

As the research field combining seagrass ecology and marine archaeology is new, and because much archaeological literature is not captured by the Web of Science and/or in non-English language (see further discussion of this aspect later), a search in Web of Science using the terms “seagrass” and “archaeology” yielded only 2 hits and none of which reported archaeological artefacts in seagrass meadows. In order to review and identify examples of the role of seagrasses in protecting marine archaeology, we therefore had to rely on direct queries to the archaeological community and we approached Danish, Mediterranean, US and Australian archaeological communities through our existing network and additional inquiries guided by the archaeologists we contacted.

**Fig. 1:**
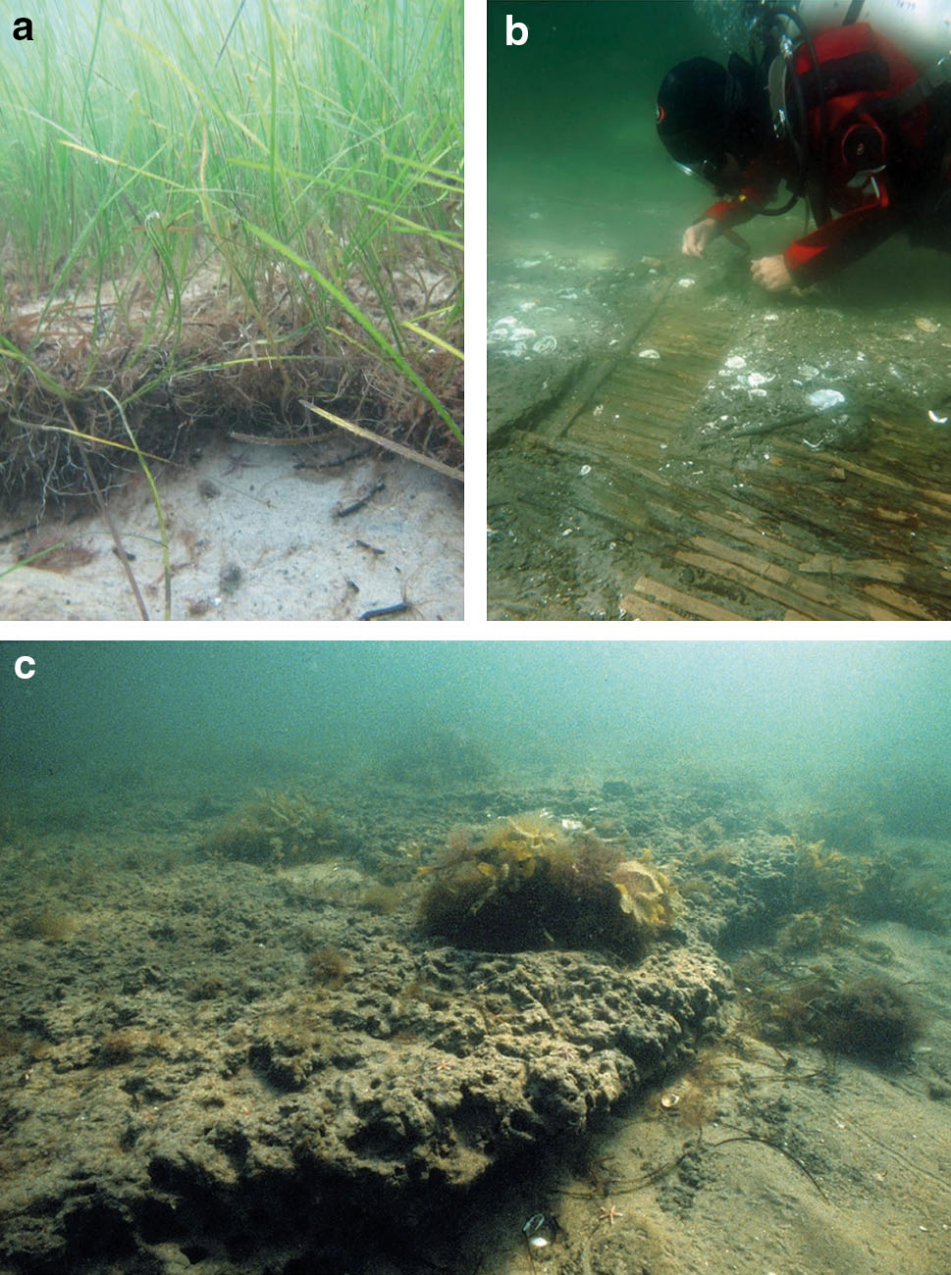
**a** Seagrass meadow currently growing on the site of Nekselø, the roots of which are preventing removal of the sand overlying the site by underwater currents. **b** Diver investigating the remains of the wattle mats from Nekselø. **c** Areas of the seabed around the fish weir site of Nekselø have lost seagrass coverage resulting in the loss of overlying sand and erosion of the layers containing archaeological remains. Photos: National Museum of Denmark

**Fig. 2:**
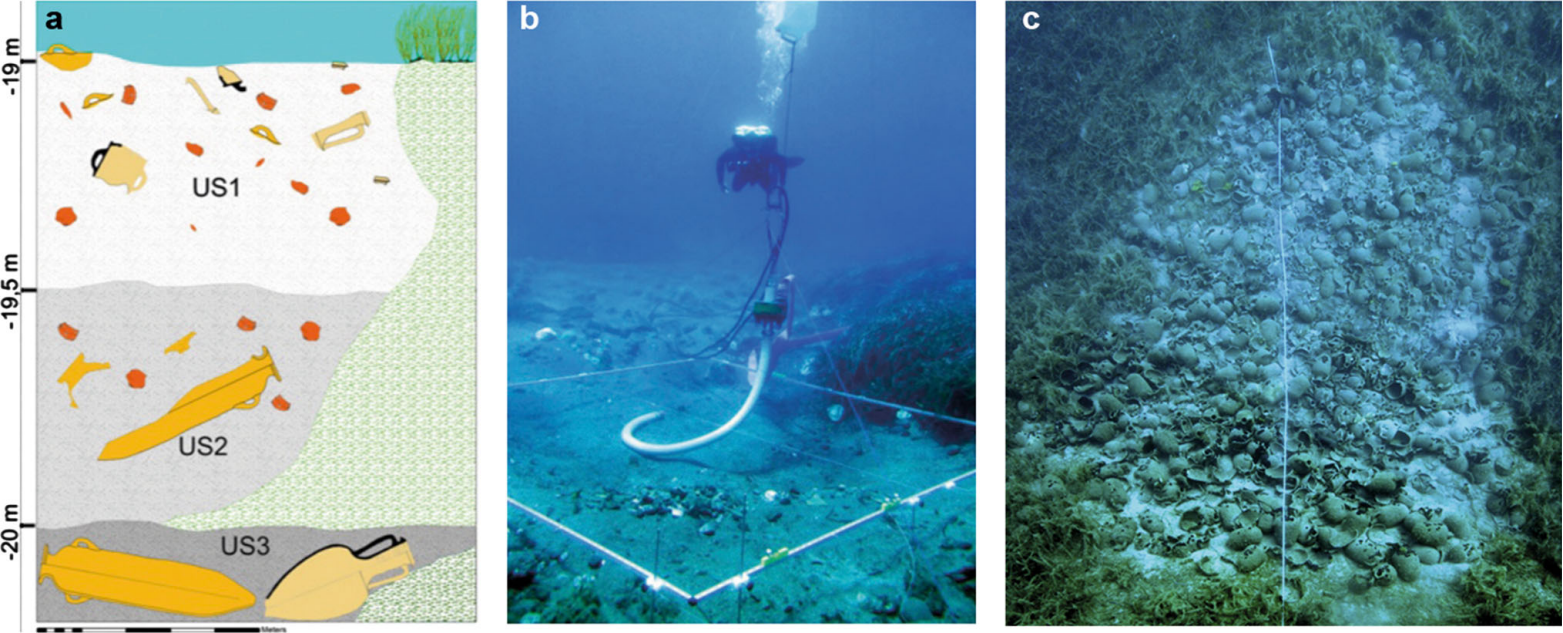
**a**, **b** Pre-Neolithic site associated with *P. oceanica* in Pantelleria Island, Central Mediterranean Sea. Multiple Punic amphores and other materials were found embedded within seagrass rhizomes in various stratigraphic units (US). This deposit was formed when the sea-level was 15 m lower than present around 7.7–9.6 cal. kyr BP. Reproduced from Abelli et al. (2016) with permission. **c** Roman amphorae from a late Roman shipwreck at − ≈32 m depth in South Prasonisi islet (Greece), site surrounded by seagrass meadows. Reproduced from Theodoulou et al. (2015) with colour version provided and permission granted from T. Theodoulou

**Fig. 3:**
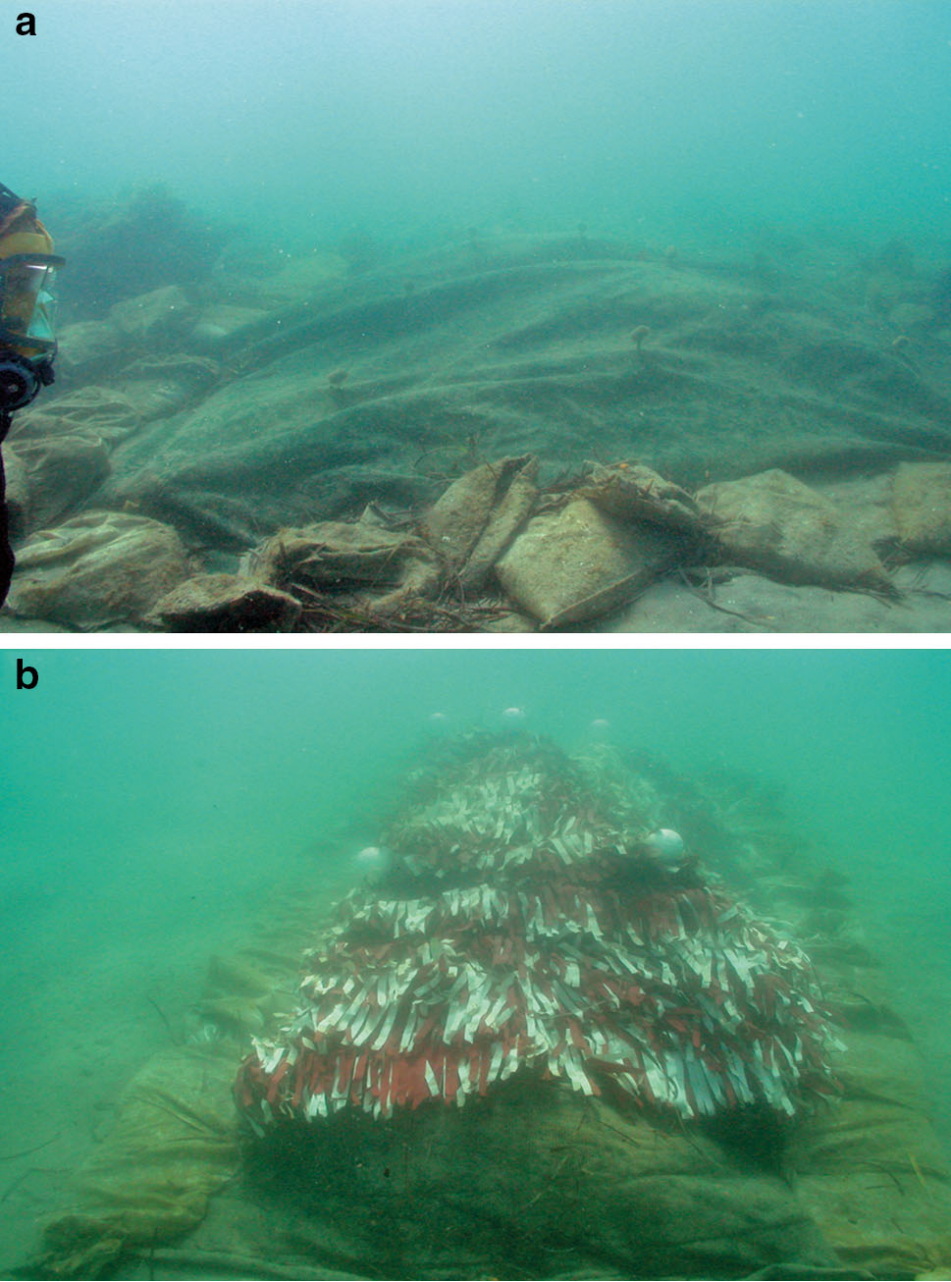
**a** The bow of the James Matthew shipwreck originally covered by seagrass and here covered by shade cloth mats held in place by sand bags; and **b** with artificial seagrass attached. Reproduced from Richards et al. (2009) with permission from the Western Australian Museum who has the copyright (details in Table S1 #18)

**Fig. 4:**
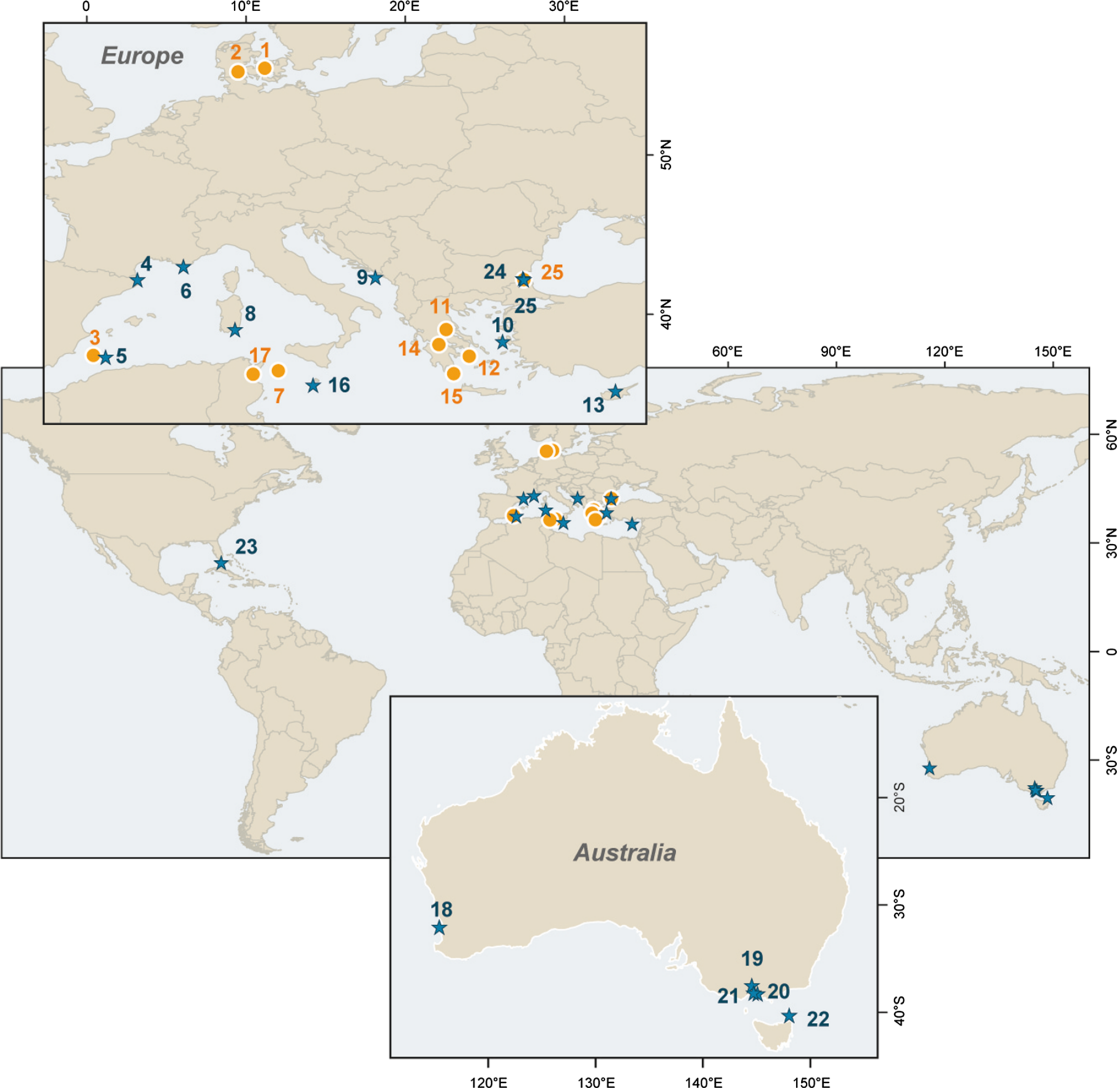
Map of 25 sites with evidence of seagrass-preserved archaeological heritage complied from the literature. Circles represent heritage from settlements, stars represent shipwrecks. For more details on sites please see Table S1. Details on site # 1 are shown in Fig. 5a

**Fig. 5:**
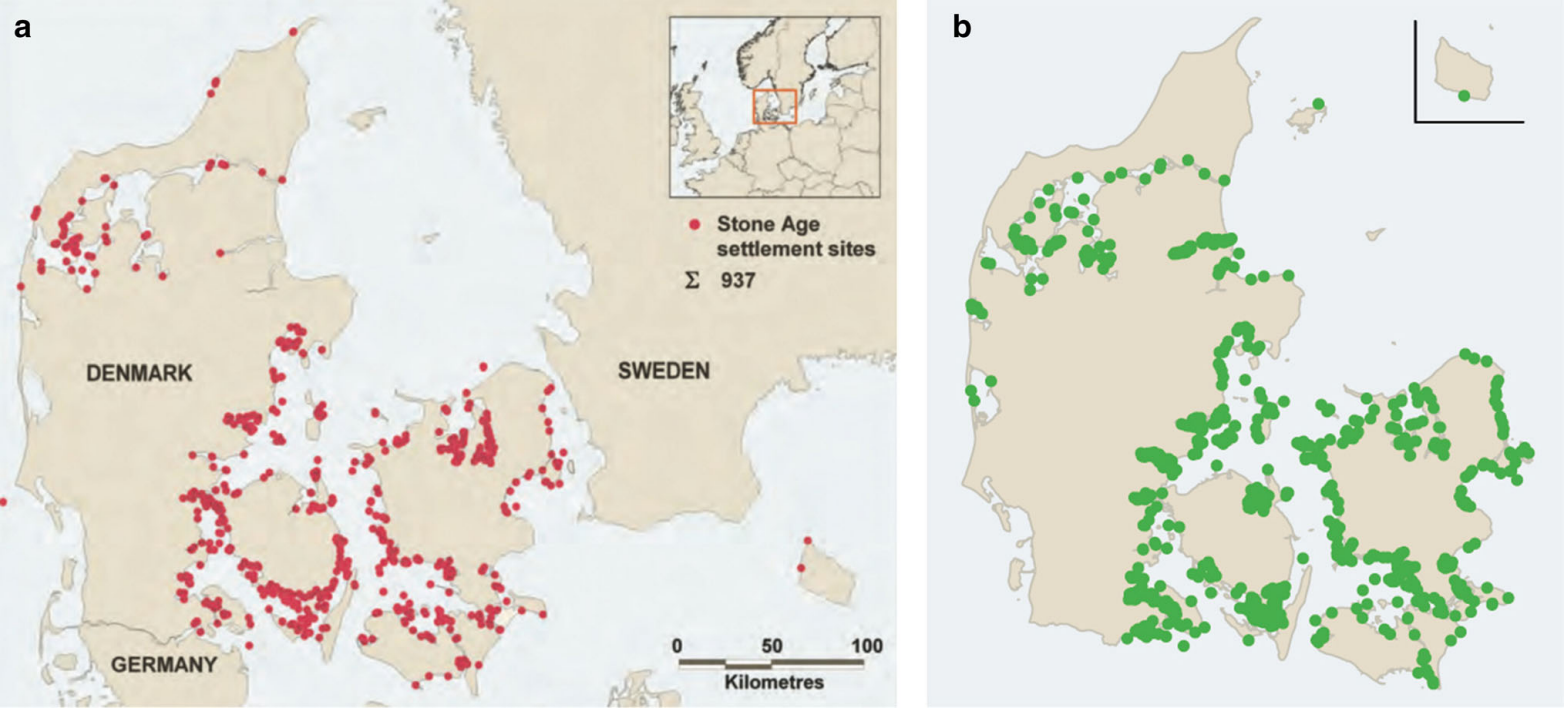
**a** Stone Age settlements from the Danish seafloor (Fischer 2011) (reproduced with permission) co-located with **b** seagrass monitoring sites in Danish coastal waters extracted from the national Danish marine database (ODA) for the period 1989–2017

The importance of seagrasses in protecting underwater human artefacts is clearly illustrated by case studies including (1) submerged prehistoric archaeological deposits protected by eelgrass in Danish coastal waters (Panel 1, Fig. 1, Fig. S1), (2) Mediterranean *P. oceanica* deposits preserving Phoenician, Greek and Roman ship wrecks, along with their cargo, over millennia (Panel 2, Fig. 2), and (3) the wreck of a former slave ship that was protected by Australian seagrass meadows until excavation disrupted the protective cover and called for intense management action to restore preservation conditions (Panel 3, Fig. 3). The evidence of the role of seagrass in preserving archaeological remains is rapidly expanding with the current review listing 25 examples across the Baltic Sea, the Mediterranean Sea, The Indian Ocean, the Gulf of Mexico and the Black Sea (Table S1, Fig. 4). This suggests that a more deliberate search may reveal that seagrass meadows worldwide protect archaeological heritage. This statement is supported by the close correspondence between the distribution of submerged prehistoric settlements in Denmark (estimated at 20 000 by the Danish Agency for Culture and Palaces) and the presence of seagrass meadows in Denmark (Fig. 5).

### Panel 1: Submerged prehistory protected by Danish seagrass meadows

Sea-level rise in the Late Pleistocene and Holocene inundated many prehistoric settlements in Denmark, resulting in the sites being waterlogged and covered by sediments overgrown by seagrass (*Zostera marina* L., eelgrass) meadows (Fig. 1a), which have provided exceptional preservation for millennia. For instance, the well-preserved Neolithic fish weir site at Nekselø (Sjælland Denmark) contains a large number of hazel wattle mats buried in sediments and is providing key evidence and insights of fishing practices and forestry management during the Neolithic (Fig. 1b, Table S1 #1). The site currently lies in 2–3 m of water in an exposed setting where the remains have only survived due to the protective cover of eelgrass meadows (Fig. 1a). Other examples are provided by the Tudse Hage and Tybrind Vig Mesolithic settlement sites, both shallow (2–3 m deep) and characterized by a rich and varied assemblage of well-preserved organic remains, such as wooden items (e.g. paddle blades with artistic decorations) human bones, including intact graves, animal bones and antler, plant food remains, and residues of charred food on pottery artefacts (Table S1 #2, Fig. S1). The well-preserved status of these sites is attributable to the combined effect of the seagrass meadows covering the sites and the anoxic conditions found within the sediments. Erosion of sediment deposits, especially from exposed, shallow settings, following loss of eelgrass meadows with the “wasting disease” in the 1930’s (e.g. Rasmussen 1973) and more recent losses related to human impact such as eutrophication have led to exposure of cultural layers, raising awareness of the archaeological heritage protected by seagrass deposits and their rapid degradation following seagrass decline ((Fischer 2011), Fig. 1c).

### Panel 2: Seagrass-preserved archaeological heritage in the Mediterranean

Multiple archaeological surveys at Cala Tramontana (Pantelleria Island, Italy) revealed several complete or fractured Punic amphorae and a few lithic artefacts below 20 m depth, which were often held by seagrass (*P. oceanica*) rhizomes ((Abelli et al. 2016), Fig. 2a and b). Predictions, based on eustatic and glacio-hydro-isostatic movements, suggest that the sea-level at the time of formation of the deposit was 15 m lower than current. The palaeolandscape reconstruction, along with archaeological evidence, date the lithic industry at Cala Tramontana back to 7.7–9.6 cal. k year BP. This represents the first trace of human visitation to Pantelleria Island, probably in order to exploit the local obsidian outcrops. Another site at around 32 m depth in South Prasonisi islet (Greece) supported an amphora workshop to transport the famous Chian wine produced in the region, the amphorae being depicted on stamps and coins of the island’s city state. This site contains a profusion of Roman amphorae from a shipwreck of the late Roman period, dated around the seventh century AD, surrounded by seagrass (*P. oceanica*) (Fig. 2c, (Theodoulou et al. 2015)). Whereas the trajectory of the seagrass meadow is unclear, the presence of invasive algae (*Caulerpa cylindracea*) and algal-covered seagrass along the edges of the site suggest decline of the meadow. This may have led to the exposure of the amphorae, which would otherwise have been damaged over time if exposed to waves and currents.

### Panel 3: Seagrass protection of a slave shipwreck in Australia

Over 7000 known shipwrecks are located around the coast of Australia. The *James Matthew* is one of the world’s best-preserved examples of a 19th century purpose-built illegal slaver. In 1973, this shipwreck was discovered underneath seagrass (*Posidonia* spp.) meadows in Western Australia, and very little was visible above the sediment prior to excavation (Table S1 #18). After excavation, the shipwreck remains were reburied with the original overburden to diminish the physical damage by organisms and hydrodynamic energy (Table S1 #18). Despite the site remaining stable and buried for many years, coastal sedimentary processes and industrial dredging activities in the immediate area are threatening this site. As a consequence, comprehensive on-site conservation surveys have been undertaken from 2000 onwards (Table S1 #18). Analyses of the surrounding sediments showed that timbers buried to a depth of ~ 30 cm were damaged by borer organisms, while timber buried below 30 cm were in good condition, informing a mitigation strategy aimed to resemble the initial preservation conditions provided by the presence of seagrasses. This strategy included sandbagging, installation of artificial seagrass mats, shade cloth mats and barriers to enhance sedimentation and achieve reburial (Fig. 3), aiming to provide preservation conditions similar to those provided by seagrasses.

The protective role of seagrass overgrowth of archaeological deposits extends beyond that of natural processes, such as oxidation and wave action, to also encompass protection from pillaging. For instance, the presence of *P. oceanica* meadows growing on top of a Phoenician shipwreck at La Manga del Mar Menor (Murcia, Spain) precluded the complete spoliation of artefacts by recreational souvenir collecting divers, who picked up obvious and diagnostic pieces but left a substantial amount of wreckage buried underneath the meadows (Polzer 2012).

## Seagrass sediment deposits as time capsules

The continuous accretion of sediments by seagrass meadows also contributes to build a millenary archive of environmental conditions (Serrano et al. 2016c), including fingerprints of human culture as documented for *Posidonia* spp. These archives can be used to reconstruct the human past, specifically millenary changes in processes such as land-use and agriculture (López-Sáez et al. 2009; López-Merino et al. 2015; López-Merino et al. 2017), mining and metallurgical activities (Serrano et al. 2011; Serrano et al. 2013; Serrano et al. 2016c), impacts of human activities on coastal ecosystems (Macreadie et al. 2012; Serrano et al. 2016d) and changes associated with colonization events by different cultures (Serrano et al. 2016c). Analyses of heavy metals along seagrass sedimentary archives have allowed identifying the impact of Greek and Roman mineral industry in the NW Mediterranean (Serrano et al. 2011; Serrano et al. 2013), and the colonization of Australia by Europeans followed by subsequent industrialization (Serrano et al. 2016c). More recently, analyses of Mediterranean seagrass rhizome tissues accumulated over time have provided evidence for the shift from chemical to digital photography through decline in silver contents (Tovar-Sánchez et al. 2010), the shift from leaded to unleaded fuel through decline in lead levels (Tovar-Sánchez et al. 2010) and the Chernobyl nuclear accident through the abundance of several radionuclides in the tissues (Calmet et al. 1991).

Whereas most interpretations of human culture from seagrass deposits have been based on heavy metal analyses, the analysis of organic materials and synthetic products provides opportunities for further reconstruction of human cultural footprints. For instance, environmental DNA (eDNA), which represents the remains of short-chain DNA fragments all organisms emit to the environment, has been recently applied to fingerprint the contributions of different macrophytes to seagrass carbon deposits (Reef et al. 2017). However, the same technique can be used to trace back ancient biodiversity, both wild and domesticated, at the time humans settled in what now are seagrass landscapes (Thomsen and Willerslev 2014; Pennisi 2015). For instance, Smith et al. (2015) used sedimentary ancient DNA analyses of coastal sediments inundated 8000 years ago to reconstruct floral and faunal changes before the inundation. This suggests that eDNA analyses of seagrass sediment archives may offer huge potentials to trace human-introduced crops and domestic animals in watersheds. Synthetic chemicals, for which the industrial nature is carefully documented, are also deposited in coastal sediments and may be used to reconstruct recent human history. It was recently suggested that the use of plastics may leave a horizon that could serve as a stratigraphic indicator of the anthropocene (Zalasiewicz et al. 2016) and, in fact, accumulations of microplastic were recently documented in sediments adjacent to *P. oceanica* meadows in the NW Mediterranean (Alomar et al. 2016).

## The cultural dimension of seagrass deposits hidden between disciplines

The account above provides compelling evidence that the value of cultural services by seagrass meadows has been grossly overlooked by ignoring the role of seagrass deposits as security vaults of underwater cultural heritage and time capsules of the human past. Whereas we do not attempt here to assign a monetary value to this service, its cultural significance is self-evident, to the extent that it should provide an important impetus for conservation and restoration.

Given the abundant evidence for the role of seagrasses in preserving the human past, it can seem surprising that this has not been highlighted before as an important cultural service of these ecosystems. This oversight is due to the different disciplines involved, including but not limited to archaeology and marine ecology, which do not share common publication platforms and even use a different vocabulary, which limits communication between these fields. For example, the term “*ecological service*” is not applied in archaeological studies, implying that reviews of seagrass services based on its use as a search term in international platforms of scientific literature (e.g. Ruiz-Frau et al. 2017) do not capture reports from the archaeological literature, even if some of them were published in English. Archaeologists do not necessarily use English as common language, which is a further impediment for communication across fields. Also, both marine archaeologists and marine ecologists have largely overlooked the role of seagrasses in protecting the human past. For instance, we only connected seagrass ecology and underwater archaeology ourselves when a marine archaeologist contacted D.K.-J. to inquire about reasons for seagrass loss in Denmark, opening the path of inquiry that led to the present review.

## Seagrass loss and conservation: Implications for the archives of the human past

Many seagrass deposits have been lost with the loss of seagrass cover (Pendleton et al. 2012; Serrano et al. 2016d) with associated risks to the preservation of archaeological heritage. Whereas most seagrass losses have been due to human impacts such as eutrophication and direct mechanical damage (Orth et al. 2006; Waycott et al. 2009), treasure hunters have also damaged seagrass meadows in attempts to pillage their associated archaeological deposits. Treasure hunters for example used a destructive technique called ‘mailboxing’ to search for gold in Spanish galleons sunk along the coast of Florida, where the galleons were overgrown by seagrass meadows. The technique involves the use of a fitting to divert propeller wash down to the seabed in order to randomly excavate seagrass sediments, leaving holes in the meadows (Varmer 1999). Controlled archaeological excavation, by contrast, involves an array of activities to systematically survey, excavate, document and preserve the sites and artefacts thereafter.

The UNESCO Convention on the Protection of the Underwater Cultural Heritage advocates in situ preservation as the preferred approach to preserving underwater archaeological sites such as shipwrecks and submerged landscapes (Maarleveld et al. 2013). Methods used on sites that have been excavated include backfilling with the removed overburden, installation of barriers, geotextiles, reburial of excavated materials, dumping sediment or placing sandbags (Staniforth and Shefi 2010; Björdal and Gregory 2012). These methods are the most cost-effective both in terms of financial investment and the time they take to deploy. They are effective in the short term and sandbags also remain effective after almost 30 years of deployment. Importantly, the methods act as good physical barriers against further erosion, generate an anaerobic environment and ensure long-term protection against continued degradation from marine biota (Gregory and Manders 2016; Pournou 2017). Artificial seagrass mats consisting of non-degradable polypropylene fronds, have, in fact, been used to simulate the protective effects of seagrass on shipwrecks, submerged prehistoric sites and other constructions. The artificial mats dampen turbulence and, hence, erosion of the sedimentary deposits, while also serving as sediment traps (Harvey 1996; Gregory and Manders 2016). However, as artificial seagrasses contribute to plastic pollution of the ocean, and lack the additional benefits, in terms of the broad suite of ecosystem services seagrass provide, natural seagrasses are preferable. Indeed, recent guidelines for the protection of underwater wooden heritage recommend seagrass restoration as an effective measure in shallow coastal waters exposed to tides and currents (Björdal and Gregory 2012). Hence, effective seagrass restoration (van Katwijk et al. 2016) is also a shared goal for further collaboration between seagrass ecologists and underwater archaeologists.

Whereas excavating a number of seagrass deposits is a predicament to advance our understanding of past human cultures, the amount of underwater archaeological sites protected by seagrasses is probably so large, with the bulk likely still to be discovered, that the vast majority of the deposits can be conserved. Likewise, the development of a reliable inventory of global seagrass extent remains a pending challenge to seagrass and Blue Carbon research. Mapping seagrass meadows should incorporate tools, such as bathymetric lidar, high-resolution multibeam sonar, dual-frequency side-scan sonar, high-resolution sub-bottom profiling and magnetometers, applied to detect artificial sub-seafloor elements such as pipelines (Tian 2008), which also hold promise for the detection of underwater archaeological heritage (Missiaen et al. 2017). Indeed, new acoustic techniques for sub-bottom imaging would allow exploration of putative underwater archaeological sites without disturbing the overlying seagrass meadows (Ward et al. 2013), thereby minimizing the damage associated with random excavation.

Realization of the role of seagrass meadows in carbon sequestration (Fourqurean et al. 2012; Duarte et al. 2013) and the risks of CO_2_ emissions with seagrass loss (Pendleton et al. 2012) have catalysed Blue Carbon strategies to mitigate and adapt to climate change through the conservation and restoration of seagrass habitats, adding to existing motivations to conserve and restore seagrass meadows. The conservation of underwater archaeological heritage is a hitherto unrealized benefit of these strategies, which may serve as an additional impetus for seagrass conservation.

In conclusion, this review of the role of seagrass deposits as security vaults of underwater archaeological heritage and time capsules/knowledge banks of the human past provides compelling evidence that the cultural services of these ecosystems have indeed been greatly overlooked. This realization provides additional motivation and benefits for Blue Carbon projects and other seagrass conservation and restoration efforts. Lastly, this review highlights the need for interdisciplinary dialogues for a comprehensive approach to the conservation of marine ecosystems. The article is particularly timely within Europe as the European Marine board and Natura 2000 are currently investigating ways of better integrating underwater cultural heritage into European maritime spatial planning (http://ec.europa.eu/environment/nature/natura2000/management/links_natural_cultural_heritage_en.htm).

## Electronic supplementary material

Below is the link to the electronic supplementary material.

Supplementary material 1 (PDF 521 kb)
