# Supplementary material for: Seagrass sedimentary deposits as security vaults and time capsules of the human past
Source: Ambio. 2018 Aug 20;48(4):325–35. doi: 10.1007/s13280-018-1083-2 (PMC6411673; doi:10.1007/s13280-018-1083-2)
Supplement: Supplementary file 1 — Supplementary material 1 (PDF 521 kb) [file 13280_2018_1083_MOESM1_ESM.pdf]

Ambio

Electronic Supplementary Material

This supplementary material has not been peer reviewed.

Title: Seagrass sedimentary deposits as security vaults and time capsules of the human past

Authors: Dorte Krause-Jensen , Oscar Serrano, Eugenia T. Apostolaki, David J. Gregory, Carlos M. Duarte

Supplementary material

Table S1. Evidence of seagrass-preserved archaeological heritage complied from the literature. References are listed below, numbered according to the numbers of the table.

**SEA**<br>**Site**	**Seagrass species**	**Archaeological evidence**
BALTIC SEA		
1. Danish coastal waters	*Z. marina*	Remains of fishing weirs and fish traps. Extensive remains from the neolithic period, which provide information about fishing practice and forestry practice.
2. Danish coastal waters	*Z. marina*	Submerged prehistoric settlement sites. Well preserved organic remains reflecting everyday life in prehistoric coastal settlements
MEDITERRANEAN SEA		
3. La Manga del Mar Menor (Spain)	*P. oceanica*	Remains of a Phoenician shipwreck.
4. Cala Culip, Cap de Creus (Spain)	*P. oceanica*	Remains of a shipwreck from the 13th to 14th Centuries AD
5. Mazarron (Spain)	*P. oceanica*	Phoenician shipwreck from the second half of the seventh century BC (part of the interior was sealed with a thick 10-15 cm layer of seagrass)
6. Gulf of Giens (Var, France)	*P. oceanica*	Roman wreck sank in about 50 or 60 BC is covered with 2 meters of seagrass matte
7. Pantelleria Island, C. Mediterranean (Italy)	*P. oceanica*	Pre-Neolithic site with punic amphorae and lithic artefacts

8. Cape Carbonara, Cavoli Island (Italy)	*P. oceanica*	Shipwrecks from late Middle Age to Modern Era 15th CE. Remains covered by seagrass with "powerful roots", reaching a matte thickness of almost 2 m
9. Cavtat, Croatia, Adriatic Sea	*P. oceanica*	Modern shipwreck (post-medieval) with amphorae for transporting oil from North Africa and wine. Seagrass meadow grows around the findings.
10. Southern Prasonisi islet, Aegean Sea (Greece)	*P. oceanica*	Roman (Late Roman 1) amphorae from shipwreck surrounded by seagrass (the best preserved and most informative shipwreck out of six in the area)
11. Metohi, Pagasetikos Gulf (Greece)	*P. oceanica*	Prehistoric Middle Bronze Age (2000-1650 BC) settlement surrounded by seagrass acting as a wave barrier
12. Cape Sounion, Aegean Sea (Greece)	*P. oceanica*	Remains of coastal installations in shallow waters within a complex seafloor composed of sandy to rocky substrate often covered by seagrass. Site of major archaeology surveys since cape Sounion served as a navigational landmark and a naval fortress protecting the sea-lanes towards the metropolis of Athens and the silver mines of Lavreotiki during the Classical and Hellenistic periods.
13. Kyrenia (Cyprus)	*P. oceanica*	Hellenistic shipwreck sank between 295-285 BC

		emerging from a carpet of seagrass *P. oceanica*
14. Corinth (Greece)	*P. oceanica*	Early Bronze Age settlement
15. Vatika Bay (Greece)		Submerged ancient Grecian city<br>Streets and monuments of ancient Neapolis, a vast 1,700 year old Roman settlement destroyed by a tsunami was recently discovered under seagrass meadows off the coast of Tunisia after several years of archaeological exploration.
16. Mellieha Bay (Malta)	*P. oceanica*	Remains of a mortar shipwreck embedded at 4 m depth within seagrass sediments.
17. Neapolis (Tunisia)	*P. oceanica*	Underwater ruins of lost Roman city, ancient Neapolis, destroyed by a tsunami, discovered under seagrass meadows
INDIAN OCEAN		
18. Cockburn Sound (Perth, Australia)	*P. sinuosa, P. australis, Amphibolis* spp.	Remains of the former slave ship James Matthew travelling from Europe to Australia (1841)
19. Port Philip Bay (Victoria, Australia)	*P. australis and/or Zostera* spp.	William Salthouse, wrecked 1841. Charts suggest shallower sea bed, earlier likely covered by seagrass. Artificial seagrass is now applied for preservation.

20. Phillip Island (Victoria, Australia)	*Amphibolis* spp, *Heterozostera* spp., *Zostera* spp. *Halophila* spp.	British inter-colonial merchant sailor and trader Leven Lass shipwreck (1854)
21. Port Philip Bay (Victoria, Australia)	*P. australis and/or Zostera* spp.	*Clarence* shipwreck, stranded in 1850. Lies on a sandy seabed in 4 to 5 meters of water. The area has seagrass beds around, but not on, the wreck site. Artificial seagrass is now applied for preservation.
22. Preservation Island (Tasmania, Australia)	*P. australis*	Sydney Cove (Eighteenth century shipwreck)
GULF OF MEXICO		
23. San Pedro, Florida Keys, US		Several Spanish plate fleet wrecks in the Florida Keys are surrounded by seagrass communities (e.g., San Pedro)
BLACK SEA		
24. Chernomorets Bay (Bulgaria)	*Z. marina*	Shipwreck with seagrass growing on top of it
25. Ropotamo (Bulgaria)	*Z. marina*, *Z. noltii*	Evidence of an Early Bronze Age settlement, an Archaic Greek trading post, an Early Byzantine harbour settlement, a Medieval anchorage and the

	traces of a possible Ottoman Era shipwreck (4th millennium BC through to the 18th century AD), an ancient settlement submerged and overgrown by seagrass

Table S1 references

1. Fischer A. 2011 Stone Age on the Continental Shelf: an eroding resource. In Submerged prehistory (eds J Benjamin, C Bonsall, C Pickard, A Fischer), pp. 298–310. Oxford: Oxbow Books.

   Pedersen L, Fischer A, Gregory D. 2017. Fletværket ved Nekselø – skovdrift og storstilet fiskeri i bondestenalderen. In P.K. Madsen (ed) Nationalmuseets Arbejdesmark, The National Museum of Denmark, Copenhagen.

2. Andersen SH. 2013. Tybrind Vig. Submerged Mesolithic settlements in Denmark Jutland Archaeological Society Publications. Vol. 77, Moesgård Museum, Århus.

3. Polzer ME. 2012 Strategies for Underwater Cultural heritage : The Case for the Bajo de la Campana Phoenician shipwreck. Gobierno de España.

4. Palou H, Reith E, Izaguirre M, *et al.* 1998. Excavacions arqueologiques subaquatiques a Cala Culip 2, Culip VI. *Monografies del CASC* I: 312-313. *In* Museu d'Arqueologia da Catalunya, Centre d'Arqueologia Subaquatica de Catalunya, Girona. 1998. NPG, ISBN 84-393-4655-7, 265 p.

5. Negueruela Martinez I. 2014. The Phoenician Ships of Mazarron. In Assyria to Iberia at the Dawn of the Classical Age. Graff SB, Rakic Y (Eds). The Metropolitan Museum of Art, New York, distributed by Yale Univeristy Press, New Haven and London.

6. Tchernia A, Pomey P, Hesnard A, *et al.* 1978. L'épave romaine de la Madrague de Giens (Var) (Campagnes 1972-1975). Fouilles de l'Institut d'archéologie méditerranéenne. *Gallia, suppl.* **24**: 1-122 + 42 pl. h.t.

7. Abelli L, Agosto MV, Casalbore D, Romagnoli C, Bosman A, Antonioli F, Pierdomenico M, Sposato A, Chiocci FL. 2016 Marine geological and archaeological evidence of a possible pre-Neolithic site in Pantelleria Island, Central Mediterranean Sea. Geol. Soc. London, Spec. Publ. 411, 97–110. (doi:10.1144/SP411.6)

8. Martin- Bueno M, Tafalla JA. 1992. Remains of a Fifteenth-Century Spanish Ship Found in Sardinia. In Underwater Archaeology Proceedings from the Society for Historical Archaeology Conference. Keith DH, Carrel TL (Eds). Society for Historical Archaeology, Kingston, Jamaica 1992.

9. Demetriou A. 2013. Management of Ancient Shipwrecks: the case of Cyprus. Report for the 2013 Honor Frost Foundation Grant. And references therein.

10. Theodoulou T, Foley B, Kourkoumelis D, Preka-Alexandri K. 2015 Roman amphora cargoes in the sea of Chios- the 2008 mission. In *Per Terram Per Mare: seaborne trade and the distribution of Roman amphorae in the Mediterranean* (ed S Demesticha), pp. 41–54. Uppsala: Åströms förlag.

11. Spondylis E, Michalis M. 2016. D22.1 Results of Test Case Greece (2014, 2015). Innovation Technologies and Application for Coastal Archaeological sites (ITACA). FP7-SPACE-2013.

12. Papatheodorou G, Geraga M, Christodoulou D, *et al.* 2014. A marine geoarchaeological survey, Cape Sounion, Greece: Preliminary results. *Mediterranean Archaeology and Archaeometry* **14**: 357-371.

13. Katzev SW. 2007. The Ancient Ship of Kyrenia, Beneath Cyprus Seas. In Valavanis P, Hardy D (*Eds*) *Great Moments in Greek Archaeology*. Oxford University Press, p 286-99.

14. Soter S, Katsonopoulou D. 2011 Submergence and uplift of settlements in the area of Helike, Greece, from the Early Bronze Age to late antiquity. *Geoarchaeology* **26**, 584–610. (doi:10.1002/gea.20366)

15. https://mission-blue.org/2017/04/vatika-bay-hope-spot-submerged-ancient-grecian-city-abuts-marine-abundance/.

16. Frost H. 1969 The mortar wreck in Mellieha Bay. In *The Gollcher Foundation, Archaeological series I*, p. vi+38. London: Apptron Press, Ltd.

17. http://www.independent.co.uk/news/world/middle-east/roman-city-discovered-underwater-ruins-tunisia-tsunami-neapolis-nabuel-cathage-a7924181.html.

18. Henderson GJ, Baker PE. 1979. James Matthews excavation. A second interim report. *Int J Naut Archaeol* **8**: 225-244.

Richards V, Godfrey I, Blanchette R *et al.* 2009. In-Situ monitoring and stabilisation of the James Matthews site. *Proceedings of the 10th ICOM Group on Wet Organic Archaeological Materials Conference, Amsterdam, 10–15 September 2007*: 113-160. Rijksdienst voor Archeologie, Cultuurlandschap en Monumenten (RACM), Amersfoort.

Richards VL. 2011. In Situ Preservation and Reburial of the Ex-Slave Ship James Matthew. *AICCM Bulletin* **32**: 33-43.

19. Harvey P. 1996 A Review of Stabilization Work on the Wreck of the William Salthouse in Port Phillip Bay. *Bull. Australas. Inst. Marit. Archaeol.* **20**, 1–8.

20. Flinders University Maritime Archaeology Program -°©- Leven Lass Historic Shipwreck -°©- 2015 Technical Report.

21. https://www.ahspp.org.au/clarence/environment/.

22. Nash M. 2009. Sydney Cove: The History and Archaeology of an Eight- teenth-Century Shipwreck. Navarine Publishing, Hobart, p. 225.

23. http://www.museumsinthesea.com/sanpedro/index.htm.

24. http://www.cua-sozopol.com/en/.

25. http://www.cua-sozopol.com/en/index.php/proekti/black-sea-map/ropotamo-2017.

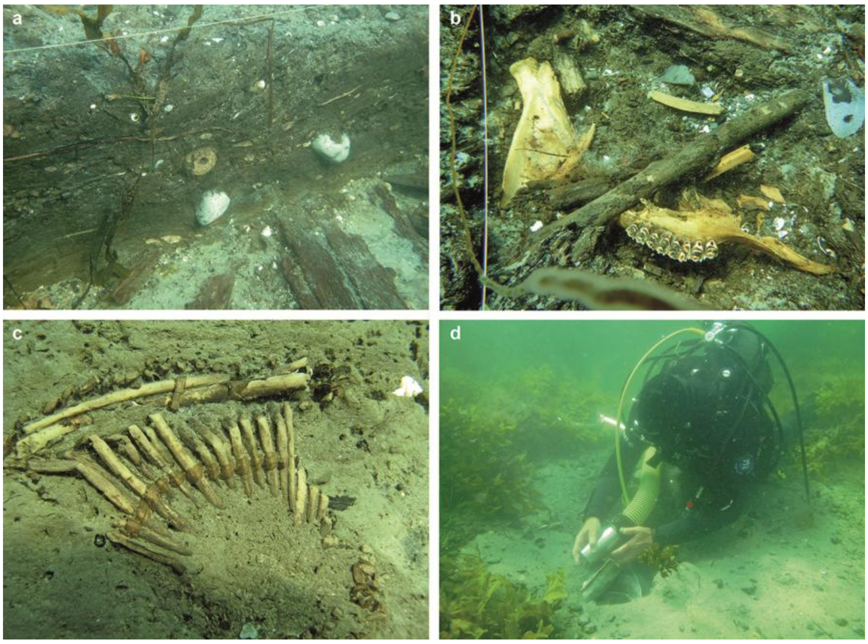

Fig. S1. The richness of finds in Tybrind Vig and Tudsehage, Denmark. (a) Stratigraphic profile of the Tudse Hage site. Anoxic organic rich sediments have preserved a wealth of organic material relating to the everyday life of people. (b) Animal remains and flint tools from the Tudse Hage site. (c) Remains of the basket at the end of a fish trap/weir. (d) Diver starting excavation of the Tudse Hage site, removing overlying sandy sediments to reveal the cultural layers. Photos: David Gregory / National Museum of Denmark.
